# Race/Ethnicity Is Not Independently Associated with Risk of Adverse Waitlist Removal among Patients with HCC Exception Points

**DOI:** 10.3390/jcm10245826

**Published:** 2021-12-13

**Authors:** Daniela Goyes, John Paul Nsubuga, Esli Medina-Morales, Romelia Barba, Vilas Patwardhan, Behnam Saberi, Zachary Fricker, Alan Bonder

**Affiliations:** 1 Department of Medicine, Loyola Medicine—MacNeal Hospital, Berwyn, IL 60402, USA; daniela.goyesvaca@luhs.org; 2 Department of Medicine, Beth Israel Deaconess Medical Center, Boston, MA 02215, USA; jnsubuga@bidmc.harvard.edu; 3 Division of Gastroenterology, Hepatology and Nutrition, Beth Israel Deaconess Medical Center, Boston, MA 02215, USA; jemedina@bidmc.harvard.edu (E.M.-M.); rbarbabe@bidmc.harvard.edu (R.B.); vpatward@bidmc.harvard.edu (V.P.); bsaberi@bidmc.harvard.edu (B.S.); zfricker@bidmc.harvard.edu (Z.F.)

**Keywords:** disparities, liver cancer, allocation

## Abstract

(1) Background: Since 2015, exception points have been awarded to appropriate candidates after six months of waitlist time to allow more equitable access to liver transplants regardless of hepatocellular carcinoma status. However, it remains unknown whether racial disparities in outcomes among waitlisted patients remain after the introduction of a 6-month waiting period for exception points. (2) Methods: Using the United Network for Organ Sharing database, we identified 2311 patients diagnosed with hepatocellular carcinoma listed for liver transplant who received exception points from 2015 to 2019. The outcome of interest was waitlist survival defined as the composite outcome of death or removal for clinical deterioration. Competing risk analysis was used to identify factors associated with death or removal for clinical deterioration. The final model adjusted for age, sex, race/ethnicity, blood type, diabetes, obesity, laboratory MELD score, tumor size, AFP, locoregional therapies, UNOS region, and college education. (3) Results: No difference was found in the risk of adverse waitlist removal among ethnic/racial groups.

## 1. Introduction

Hepatocellular carcinoma (HCC) is one of the leading causes of cancer-related mortality in the United States [1]. Its incidence has doubled in recent decades, especially in Black and Hispanic patients who often require liver transplants (LT) [2]. Racial/ethnic disparities in overall survival in patients with HCC have been reported [3]. These inequalities include, but are not limited to, more advanced stages at diagnosis [4] and lower rates of living donor liver transplantation [5], ablation, and resection [6], with the greatest impact on Black patients. To grant timely liver transplantation, MELD-exception points are available to patients with HCC. Since 2015, exception points have been awarded to appropriate candidates after six months of waitlist time [7]. This mandatory 6-month waiting period has allowed more equitable access to LT regardless of HCC status [8]. However, it remained unknown whether racial disparities in outcomes among waitlisted patients remain after the introduction of a 6-month waiting period for exception points. Therefore, we aimed to compare the rate of adverse waitlist removal among Caucasian, Black, and Hispanic patients with HCC-exception points from 2015–2019.

## 2. Materials and Methods

### 2.1. Study Population

Using the United Network for Organ Sharing (UNOS) database, we identified all patients diagnosed with HCC listed for LT who received exception points from 8 October 2015 to 8 October 2019. The study population was limited to those transplanted after the implementation of the policy of a mandatory 6-month waiting period. We excluded: (1) children (<18 years old); (2) recipients of live donor transplants; (3) multiple-organ transplants; and (4) those who had a history of a previous liver transplant. Race/ethnicity was identified by the patients’ self-reported history provided when registering on the waitlist and categorized as Caucasian, Black, or Hispanic. This study was exempt from institutional review board approval.

### 2.2. Outcome

The outcome of interest was waitlist survival, defined as the composite outcome of death or removal for clinical deterioration (UNOS removal codes 5, 8 and 13). We compared waitlist survival among groups using competing risk analysis with liver transplantation as a competing risk.

### 2.3. Study Variables

We collected demographic data including sex and age, as well as clinical characteristics at listing such as body mass index (BMI), blood type, etiology of liver disease, history of diabetes, obesity, ascites, encephalopathy, laboratory MELD score, time spent on the waitlist, UNOS region, and college education. Tumor characteristics at listing, including tumor size and alpha-fetoprotein (AFP) level, were also included in the data.

The etiology of the underlying liver disease was extracted from the secondary diagnosis codes. Categories included non-alcoholic steatohepatitis (NASH), alcohol-related liver disease (ALD), hepatitis B virus (HBV), hepatitis C virus (HCV), cholestatic liver disease, and autoimmune hepatitis (AIH). Obese patients (BMI ≥ 30 kg/m^2^) with cryptogenic cirrhosis were categorized as NASH [9]. Patients with HCV and ALD were categorized as HCV [10]. Patients with primary biliary cholangitis, secondary biliary cirrhosis, and primary sclerosing cholangitis were categorized as cholestatic liver disease [11].

### 2.4. Statistical Analysis

Recipient race/ethnicity was used to stratify clinical and demographic characteristics. Continuous variables that were not normally distributed were reported as the median and interquartile range (IQR) and were compared using the Kruskal–Wallis test. Categorical variables were summarized using percentages and compared using Pearson’s chi-square test (χ2).

Competing risk analysis was used to identify factors associated with death or removal for clinical deterioration. Univariate analysis was performed for each variable to determine which covariates would be included in the adjusted model. Variables with a *p* < 0.10 in the univariate analysis as well as those deemed to be of clinical significance by the investigators were included in the model. Patients with incomplete data were excluded from the multivariable analysis (Table A1). The final models were adjusted for age, sex, race/ethnicity, blood type, diabetes, obesity, laboratory MELD score, tumor size, AFP, UNOS region, locoregional therapies, and college education. We reported adjusted associations of covariates and overall survival as the sub-distribution hazard ratio (SHR) and 95% confidence intervals (CI). Analyses were performed using Stata version 14.0 (College Station, TX, USA; StataCorp LP).

## 3. Results

### 3.1. Characteristics of the Population: Descriptive Statistics

We identified 4798 patients with a diagnosis of HCC and approved exception points. We then excluded 2487 patients due to an unacceptable amount of missing data. Our final population numbered 2311, including 1645 (71%) Caucasian, 217 (9%) Black, and 449 (19%) Hispanic patients. The baseline patient characteristics are detailed in Table 1. A greater proportion of Hispanic recipients had diabetes (48%). Cirrhosis complications, such as encephalopathy and ascites, were less common among Black patients. Across the groups, HCV was the most prevalent etiology among patients undergoing liver transplantation.

In accordance with regional demographics, a greater proportion of Caucasian patients were transplanted in regions 3 and 11. Black patients were more frequently transplanted in regions 2 and 3, and Hispanic patients in regions 4 and 5. Hispanic recipients experienced a longer median waitlist time, 292 days (IQR 229-433), compared to other groups waiting to undergo liver transplants.

### 3.2. Waitlist Survival: Analytical Statistics

**Figure 1:**
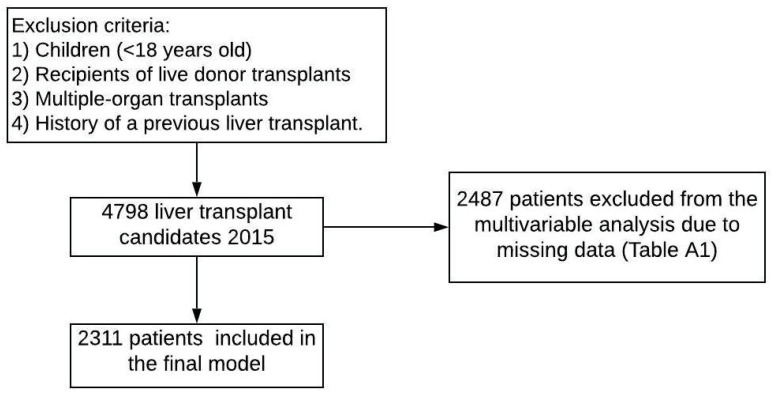
Flowchart of exclusion criteria.

**Figure 2:**
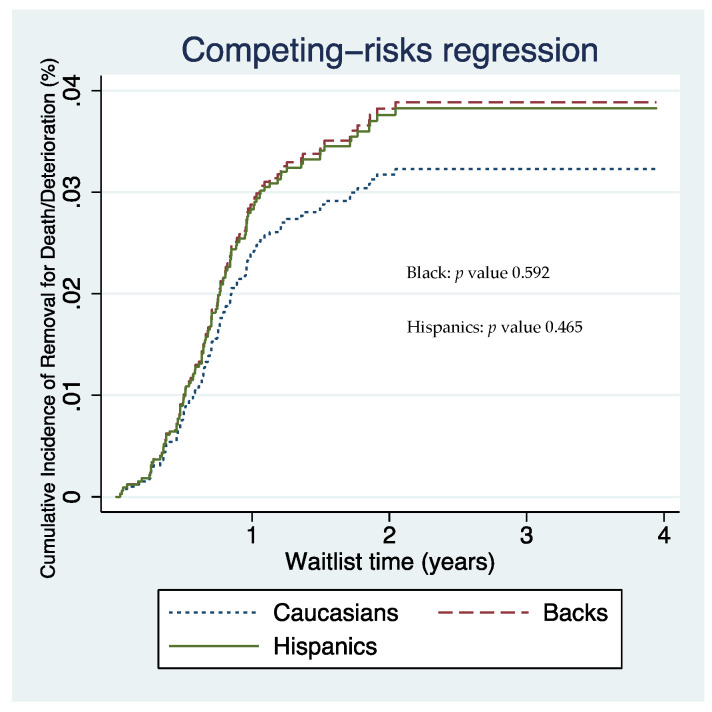
Competing risk regression of Caucasian, Black, and Hispanic patients demonstrating relative removal from the waitlist for death or clinical deterioration.

Our multivariate competing risk analysis (adjusted for age, sex, race/ethnicity, blood type, diabetes, obesity, MELD score, tumor size in cm, AFP, UNOS region, locoregional therapies, and college education) included 2311 complete cases (Figure 1). There was no difference in the risk of adverse waitlist removal among ethnic/racial groups (Table 2) (Figure 2).

## 4. Discussion

Using the UNOS database in this study, we sought to compare the rate of adverse waitlist removal between Caucasian, Black, and Hispanic patients with HCC exception points following the adoption of the 6-month mandatory wait time. We found that despite overall disparities in the stage at presentation [4,6], Black and Hispanic patients who proceed to waitlisting with HCC exception points are not at increased risk of being removed from the waitlist because of death or medical unsuitability for a transplant relative to Caucasian patients. To our knowledge, this is the first study specifically investigating the presence of racial disparities in this population.

Our results coincide with prior literature that has also highlighted the lack of disparities in the transplant waitlist outcomes in patients with HCC. For example, using data from the UNOS database, Moylan et al. show that Black patients with HCC do not have an increased likelihood of death or becoming too sick for LT [12], despite higher MELD at listing. Likewise, single-center experience from 2007 to 2009 shows comparable rates of transplantation among waitlisted patients (Black candidates 27%, Caucasian candidates 28%) [13].

There are several potential explanations for the absence of disparities once patients receive exception points. For instance, studies have shown that the 6-month mandatory waiting period may reduce subsequent disparity related to the stage of disease at presentation due to increased waitlist removal for progressive HCC for patients presenting with a more advanced disease [14]. It is possible that the highest impact is felt by racial/ethnic minorities who may present with a more advanced disease [5]. For example, Black patients were found to be more likely to have Barcelona Clinic Liver Cancer stage C (24% vs. 15%, *p* = 0.010) and multifocal tumors (39% vs. 32%, *p* = 0.014) compared to Caucasian patients [15], possibly increasing the risk of waitlist dropout prior to receiving exception points. Furthermore, significant resources available to patients waitlisted for transplant at most centers may help to mitigate any socioeconomic disparities related to access to care suffered by Black or Hispanic patients.

On the other hand, while the 6-month waiting period does not affect mortality rates once patients are listed, racial and ethnic disparities prior to waitlist placement continue to have a profound negative impact on access to LT [16]. These disparities are likely due to various factors such as socioeconomic status [17], insurance status [18], and delayed referrals to transplant centers [19]. Therefore, our study population may underestimate the dropout rates among racial/ethnic minorities.

The strengths of our study include the use of a well-characterized, nationwide database of transplant candidates, allowing our results to be generalizable to most US transplant centers. Additionally, the use of time-to-event analysis and competing risk analysis allowed for simultaneous assessment of the effects of competing risks, such as waitlist removal for death or deterioration and transplant, on the survival probability for each failure type. However, this study is limited by the retrospective nature of the analysis and the absence of details, such as tobacco use, that may influence outcomes. Race/ethnicity was self-reported, potentially introducing the risk of misclassification bias. Lastly, although we included all available data from a nationwide database, it is possible that a larger sample size might permit the identification of a statistically significant difference between groups.

## 5. Conclusions

Since the introduction of a mandatory 6-month waiting period for HCC exception points, there is no apparent difference in the risk of adverse waitlist dropout between Black, Hispanic, and Caucasian patients. However, important work is needed to improve disparities in access to transplant listing. This remains an area for improvement in the provision of equitable care for all patients with advanced liver disease.

## Figures and Tables

**Table 1:** Baseline demographic and clinical characteristics of patients diagnosed with HCC who had exception points from 2015 to 2019, grouped according to ethnicity (*n* = 2311).

	Descriptive Statistic		
Variables	Caucasian *n* = 1645 (71)	Black *n* = 217 (9)	Hispanic *n* = 449 (19)
Age, median (IQR)	62 (59–66)	62 (59–65)	61 (56–66)
Sex (male), *n* (%)	1345 (82)	154 (71)	326 (73)
BMI, median (IQR)	29 (26–33)	29 (25–33)	30 (26–33)
Blood type, *n* (%)			
A	693 (42)	50 (23)	154 (33)
B	158 (10)	49 (23)	40 (9)
AB	58 (4)	8 (4)	6 (1)
O	736 (45)	110 (51)	249 (55)
Diabetes, *n* (%)	562 (34)	73 (34)	215 (48)
Obesity, *n* (%)	699 (42)	89 (41)	214 (48)
Ascites, *n* (%)			
Absent	1050 (64)	171 (79)	274 (61)
Slight	527 (32)	39 (18)	151 (34)
Moderate	67 (4)	7 (3)	24 (5)
Encephalopathy, *n* (%)			
None	1176 (72)	175 (81)	306 (68)
Grade 1–2	451 (27)	42 (19)	141 (31)
Grade 3–4	17 (1)	0 (0)	2 (0.45)
Underlying liver disease, *n* (%)			
HCV	893 (64)	156 (84)	207 (54)
ALD	204 (15)	15 (8)	85 (22)
HBV	25 (2)	7 (4)	2 (1)
NASH	243 (17)	2 (1)	82 (21)
Cholestatic liver disease	15 (1)	3 (2)	5 (1)
AIH	15 (1)	2 (1)	5 (1)
MELD score at listing, median (IQR)	9 (7–11)	9 (7–11)	10 (8–12)
UNOS region, *n* (%)			
1	57 (3)	10 (5)	7 (2)
2	178 (11)	31 (14)	23 (5)
3	282 (17)	45 (21)	66 (15)
4	142 (9)	28 (13)	125 (28)
5	171 (10)	13 (6)	126 (28)
6	95 (6)	2 (1)	17 (4)
7	143 (9)	26 (12)	24 (5)
8	119 (7)	7 (3)	13 (3)
9	60 (4)	17 (8)	26 (6)
10	164 (10)	15 (7)	9 (2)
11	234 (14)	23 (11)	13 (3)
Days on waitlist, median (IQR)	244 (205–326)	257 (209–357)	290 (232–434)
AFP, (ng/mL) median (IQR)	6 (4–12)	9 (5–23)	7 (4–17)
Tumor size (cm), median (IQR)	2.6 (2.1–3.5)	2.7 (2.1–3.6)	2.8 (2.2–3.8)
Locoregional therapies, *n* (%)			
Transarterial chemoembolization	1078 (66)	148 (68)	316 (70)
Chemical ablation	46 (3)	5 (2)	15 (3)
External beam radiation	54 (3)	2 (1)	8 (2)
Radiation microspheres	296 (18)	39 (18)	69 (15)
Thermal ablation	529 (32)	69 (32)	143 (32)
Non-college education	799 (49)	128 (59)	324 (72)

BMI—body mass index; HBV—hepatitis B virus; HCV—hepatitis C virus; ALD—alcohol-related liver disease; NASH—nonalcoholic steatohepatitis; AIH—autoimmune hepatitis; MELD—model for end-stage liver disease; UNOS—United Network for Organ Sharing; AFP—alpha-fetoprotein; IQR—interquartile range.

**Table 2:** Multivariable analysis.

Multivariable Analysis			
Variables	SHR *	95% CI *	*p* Value *
Age	1.01	0.98–1.05	0.275
Sex (male)	0.98	0.62–1.55	0.934
Blood type			
O	Ref		
A	1.19	0.80–1.79	0.379
B	0.39	0.15–0.99	0.049
AB	1	0.29–3.39	0.995
Diabetes	1.04	0.70–1.55	0.815
Obesity	1.03	0.69–1.53	0.874
MELD score at listing	1.1	1.07–1.15	<0.001
Ethnicity			
Caucasian	Ref		
Black	1.20	0.60–2.41	0.592
Hispanic	1.18	0.74–1.88	0.465
Tumor size	1.06	1.01–1.12	0.019
Alpha-Feto Protein	1	0.99–1	0.196
College Education	1.35	0.89–2.03	0.150

SHR—sub-distribution hazard ratio; Ref—reference; MELD—model for end-stage liver disease; CI—confidence interval. * Multivariable analysis adjusted for all variables in this table, as well as UNOS region and locoregional therapies.

## Data Availability

Data can be requested at https://unos.org/data/.

## Appendix A

**Table A1:** Missing data stratified by race/ethnicity.

Variable Missing	Caucasian = 2034	Black = 251	Hispanic = 570
Diabetes, *n* (%)	6 (0.2)	0 (0)	7 (0.8)
AFP, *n* (%)	552 (16)	69 (16)	174 (19)
Tumor size, *n* (%)	1259 (37)	147 (34)	318 (34)
College, *n* (%)	71 (2)	13 (3)	23 (2)
Locoregional therapies	146 (8)	22 (9)	48 (10)

AFP—alpha-fetoprotein.
